# Genetic Exchange of Multidrug Efflux Pumps among Two Enterobacterial Species with Distinctive Ecological Niches

**DOI:** 10.3390/ijms10020629

**Published:** 2009-02-19

**Authors:** Nehaya Al-Karablieh, Helge Weingart, Matthias S. Ullrich

**Affiliations:** School of Engineering and Science, Jacobs University Bremen, Campus Ring 1, 28759 Bremen, Germany; E-Mails: n.alkarablieh@jacobs-university.de (N.K.); h.weingart@jacobs-university.de (H.W.)

**Keywords:** *Erwinia amylovora*, *Escherichia coli*, TolC, AcrAB, multidrug efflux

## Abstract

AcrAB-TolC is the major multidrug efflux system in *Enterobacteriaceae* recognizing structurally unrelated molecules including antibiotics, dyes, and detergents. Additionally, in *Escherichia coli* it mediates resistance to bile salts. In the plant pathogen *Erwinia amylovora* AcrAB-TolC is required for virulence and phytoalexin resistance. Exchange analysis of AcrAB-TolC was conducted by complementing mutants of both species defective in *acrB* or *tolC* with alleles from either species. The *acrB* and *tolC* mutants exhibited increased susceptibility profiles for 24 different antibiotics. All mutants were complemented with *acrAB* or *tolC*, respectively, regardless of the taxonomic origin of the alleles. Importantly, complementation of *E. amylovora* mutants with respective *E. coli* genes restored virulence on apple plants. It was concluded that AcrAB and TolC of both species could interact and that these interactions did not yield in altered functions despite the divergent ecological niches, to which *E. coli* and *E. amylovora* have adopted.

## 1. Introduction

Rising levels of bacterial multidrug resistance are an increasing problem in the treatment of infectious diseases. This phenotype is often associated with the expression of multidrug efflux (MDE) transporters, which export drugs out of the cell. The involved proteins belong to five well-characterized families: the ATP-binding cassette (ABC) family, the major facilitator superfamily (MFS), the resistance-nodulation-cell division (RND) superfamily, the small multidrug resistance (SMR) family, and the multidrug and toxic compound extrusion (MATE) family [1]. Efflux by transporters of the MFS, RND, SMR, and MATE families is driven by proton (or sodium) motive force while ATP hydrolysis drives efflux in ABC transporters [2–5].

RND-type transporters are widespread in Gram negative bacteria. In these efflux pumps, an outer membrane protein of the TolC family co-operates with both, an energized inner membrane translocase typically comprising a proton anti-porter and an adaptor protein located in the periplasmic space, thus forming a tripartite efflux system [2,4]. A remarkable feature of these efflux systems is the wide range of substrates that are recognized by individual pumps [6–7].

*Escherichia coli*, a member of *Enterobacteriaceae*, is one of the best and most thoroughly studied bacterial species. Some *E. coli* strains live as harmless commensals in the human intestines while other strains are pathogenic causing diseases and high mortality in humans [8]. AcrAB-TolC of *E. coli* is the best characterized RND efflux pump and has emerged as the principal structural and biochemical model system. The anti-porter AcrB and the membrane fusion adaptor AcrA form a translocase unit that interacts with the outer membrane protein TolC thus comprising a contiguous protein complex spanning the bacterial cell envelope and enabling drug efflux [9–10].

AcrAB-TolC mediates resistance towards a wide variety of hydrophobic and amphiphilic compounds including bile salt, detergents, dyes, and antimicrobial agents [10]. Respective alleles of different gram-negative bacteria show a high degree of similarity and their deduced amino acid sequences are homologous [11–12]. The *acrAB* loci are regulated by members of TetR family of transcriptional repressors named AcrR. The *acrR* gene is located 141 bp upstream of the *acrAB* locus and is divergently transcribed [13]. AcrB is of particular interest since it mediates substrate specificity of the tripartite MDE pump towards a wide range of structurally diverse substances [14]. AcrAB recruits the separately expressed outer membrane protein TolC to extrude substrates from the inner membrane or the cytoplasm without any substrate accumulation in the periplasmic space [15].

Homologues of *E. coli* TolC have been identified in numerous Gram-negative bacteria. Different AcrAB-like transport systems have evolved in *E. coli* and all share TolC as outer membrane partner [16–18]. The same was found for *Klebsiella pneumoniae* where AcrAB homologues specifically interact with the TolC-like outer membrane protein KocC, and the respective genes are not co-transcribed [19]. In contrast, in other gram-negative bacteria individual TolC-like channels are often unique for a given RND-type transporter and their genes are consequently co-expressed in the same gene cluster as the RND-type pumps such as in the case of the *oprJMN* cluster in *Pseudomonas aeruginosa* [20–21] or the *mtrRCDE* cluster in *Neisseria gonorrhoeae* [22]. In bacterial species other than *E. coli*, the functions attributed to TolC are not linked to a single *tolC* allele but are distributed among various *tolC* homologues [23].

Comparative genome analyses revealed high numbers of MDE pumps in soil or plant-associated bacteria [24]. Plants produce an array of diverse secondary metabolites that have antimicrobial activities including preformed so-called phyto-anticipants and phytoalexins, which are synthesized in response to pathogen attack [25–26]. An increasing number of RND-type transporters conferring multidrug resistance in plant-associated bacteria have recently been identified, for examples in *Agrobacterium tumefaciens* [27], in *Pseudomonas syringae* [28], in *Erwinia chrysanthemi* [29], in *Bradyrhizobium japonicum* [30], in *Xanthomonas oryzae* [31], and in *Ralstonia solanacearum*[32].

*E. amylovora*, a member of *Enterobacteriaceae,* is the causal agent of fire blight on apple and various other *Rosaceae*. Burse *et al*. [33] demonstrated that AcrB played an important role in virulence of *E. amylovora* and was required for resistance towards diverse plant phytoalexins as well as for successful colonization of the host plant. Recently, mutational analysis showed that TolC is also indispensable for virulence and bacterial *in planta* multiplication by mediating resistance towards phytoalexins through its exclusive interaction with AcrAB in *E. amylovora* [34].

Herein, knock-out mutants of *E. amylovora* and *E. coli* defective in *acrAB* or *tolC*, respectively, were complemented with respective homologous or heterologous alleles in order to investigate whether components of the two systems can be exchanged thereby altering substrate spectra and to determine potential ecological niche-mediated adaptations for both bacterial species.

## 2. Results and Discussion

### 2.1. Bioinformatics approach

A BLAST search with the genome sequence of *E. amylovora* Ea237 (http://www.sanger.ac.uk/projects/E.amylovora) using the amino acid sequence of AcrB from *E. coli* K12 strain DH10B (accession number YP-001729367) as query identified six homologous sequences in the genome of Ea237. At the amino acid sequence level, the respective predicted *E. amylovora* proteins showed the following identities (similarities given in brackets): AcrB with 83% (92%), AcrD with 78% (89%), MdtB with 81% (90%), MdtC with 73% (86%), and two MdtB- and MdtC-like proteins with 63% (79%) and 56% (73%), respectively [35–36].

Additionally, the respective *E. amylovora* homologues of the *E. coli* membrane fusion protein AcrA and the transcriptional repressor AcrR showed 73% and 62% identity (83% and 79% similarity), respectively [13,35]. A BLAST search using the amino acid sequence of TolC from *E. coli* revealed presence of only one TolC homologue in *E. amylovora* with 77% identity (86% similarity) suggesting a high degree of conservation of genomic arrangements between the two enterobacterial species.

### 2.2. Susceptibility of E. coli and E. amylovora towards antimicrobial compounds

The susceptibility of the wild types and respective single mutants with deletions in *acrB* or *tolC* of *E. coli* and *E. amylovora* to a variety of antimicrobial compounds were examined in complex and minimal medium using the determination of minimal inhibitory concentrations (MIC) (Tables 1–4). Double mutants defective in *acrB* and *tolC* served as negative controls. Different plant-derived antimicrobial compounds, which were previously reported to be substrates for *E. amylovora* AcrAB-TolC [33–34], bile salt as a reported substrate for AcrAB-TolC in *E. coli* [37], and various other antimicrobials were tested. Single deletion of *acrB* or *tolC* and simultaneous deletion of *acrB* and *tolC* resulted in increased susceptibility of *E. coli* mutants toward all tested plant-borne antimicrobial compounds. The respective MICs for phloretin, (+)-catechin, naringenin, quercetin, and berberine decreased more than 4-fold, 4-fold, 8-fold, 8-fold, and 16-fold, respectively, in complex medium and about 8-fold in minimal medium except for berberine, for which the MICs were reduced about 64-fold, as compared to *E. coli* wild type MICs (Tables 1 and 3). Similarly, *E. amylovora* mutants defective in *acrB* or *tolC* and the double mutant exhibited 4-fold, 8-fold, 8-fold, 16-fold, and 32-fold decreased MICs towards (+)-catechin, phloretin, naringenin, quercetin, and berberine, respectively, in both, complex and minimal medium (Tables 2 and 4). Consequently and despite of the fact that *E. coli* is neither a plant pathogen nor usually exposed to plant-borne chemical defense reactions, AcrAB-TolC of this human-associated bacterium can exclude phytoalexins. This interesting result further substantiated the broad substrate spectrum of this MDE system [14,38] and is in line with the idea that intestinal microbes are constantly challenged by toxic substances derived from plant-borne diet in mammals [39]. Alternatively, our result might indicate a high degree of phylogenetically conserved functionality of the AcrAB-TolC complex among *enterobacteriaceae*.

The three tested *E. amylovora* mutants were about 8-fold more sensitive to bile salt as compared to the wild type regardless of the used medium. In contrast, the three *E. coli* mutants exhibited a 64-fold increased sensitivity to bile salt in both media in comparison to the wild type (Tables 1–4). This result clearly demonstrated that *E. amylovora* may possess an alternative MDE pump during the detoxification process for bile salts such as demonstrated for EefABC in *Enterobacter aerogenes* [40]. It remained obscure why the *E. coli* mutants exhibited higher susceptibilities towards bile salt since one might have assumed a better adaptation of this bacterium to this toxic compound [41].

In contrast to the mutants of *E. amylovora*, growth medium-dependent susceptibility towards the following group of antimicrobials was observed for the three *E. coli* mutants: acriflavine, novobiocin, ampicillin, cefoperazone, mitomycin, tetracycline, nalidixic acid, norfloxacin, ciprofloxacin, SDS, ethidium bromide, and crystal violet. For those substances the *E. coli* wild type was about 5- to 20-fold more resistant as compared to the mutants in complex medium but only 4- to 10-fold more resistant in minimal medium (Tables 1 and 3). *E. amylovora* mutants were 5- to 40-fold more sensitive to those compounds as compared to their wild type regardless of the used growth medium (Tables 2 and 4). These apparently conflicting results might be rather due to the auxotropic status of *E. coli* TG1 and its general physiological fitness in minimal medium [42] than due to substrate specificity alterations.

The MICs of the aminoglycosides amikacin and tobramycin for the *acrB* mutants of *E. coli* and *E. amylovora* did not differ from those determined for the wild type strains suggesting that those substances did not act as substrates of AcrB. In contrast, the same substances caused 8-fold and 16-fold increased sensitivities for the *tolC* mutants and the *acrB* / *tolC* double mutants of both species in complex and minimal medium, respectively. It was previously demonstrated that TolC additionally to its function with AcrB could specifically interact with another RND-type transporter of *E. coli* termed AcrAD in extruding a variety of hydrophilic aminoglycosides from the periplasm and cytoplasm [38,43].

Additive effects in the reduction of MICs due to simultaneous disruption of *acrB* and *tolC* regardless of the used species and medium could be observed for erythromycin, rifampin, jasmone, and clotrimazole. The MIC values of the *acrB* mutants for these compounds were reduced 4- to 10-fold. In contrast, respective MIC values declined in the *tolC* mutants about 10- to 40-fold. Susceptibilities increased 64- to 200-fold in the *acrB* / *tolC* double mutant of *E. coli* and about 80-fold in the respective *E. amylovora* double mutant (Tables 1–4), suggesting that outer membrane-bound TolC might interact with additional partners during the extrusion of these compounds. Chollet *et al*. [44] demonstrated that telithromycin, a ketolide comparable to macrolides, is not a substrate for AcrAB-TolC but it is efficiently recognized by another PAβN-sensitive system pump. Elkins and Mullis [14] showed that the MFS-type tripartite system, EmrAB-TolC, of *E. coli* has the extraordinary capacity to transport mammalian steroid hormones. This transporter is ‘silent’ under normal laboratory conditions and its contribution to MDE resistance towards steroids and macrolides is masked by the overlapping substrate repertoire of the AcrAB-TolC system [14]. Likewise, MacAB of *E. coli* is cooperating with TolC in exporting macrolides [45]. The herein observed additive effects in MIC reduction may be related to presence of EmrAB in both, *E. coli* and *E. amylovora*. A BLAST search in the genome sequence of *E. amylovora* 237 revealed presence of an EmrB homolog with 83% identity and 92% similarity but no MacB homologs.

### 2.3. Allelic exchange analysis of AcrAB-TolC in E. amylovora and E. coli

As expected and in line with our previously published data [33–34], the MIC phenotypes of *acrB* and *tolC* single mutants of *E. amylovora* could be fully restored to wild type levels with clones containing *E. amylovora acrAB* and *tolC*, respectively. Without any exceptions, the same results were obtained when respective *E. coli* mutants were complemented with cloned *E. coli acrAB* and *tolC* genes (Tables 1–4). Next, single mutants of *E. amylovora* were transformed to carry *acrAB* or *tolC*, respectively, derived from *E. coli* and vice versa. Thus it was tested whether or not the individual components of the AcrAB-TolC complex from the two enterobacterial species can interact without interferences. Interestingly, all thus generated transformants showed fully restored wild type MIC phenotypes (Tables 1–4), suggesting that the AcrAB-TolC efflux systems of *E. coli* and *E. amylovora* are truly interchangeable despite of the variable ecological niches both organisms occupy. The results furthermore allowed the conclusion that the divergence in micro-ecological adaptation had not led to specialized MDE pumps with respect to AcrAB-TolC but that this system is multifunctional and ancestral. This confirmed an earlier study, in which MDE pumps from *Ralstonia solanacearum* were used to complement respective *E. coli* mutants [32]. Our data are also in agreement with those from Tikhonova *et al.* [46] and Bokma *et al*. [47], who reported on the functionality of AcrB-MexB hybrid proteins and the directed evolution-based adaptation of *E. coli* TolC to the MexAB translocase of *Pseudomonas aeruginosa*. The later authors generated active hybrid pumps by challenging a library of mutated and shuffled TolC variants to adapt to the non-cognate *P. aeruginosa* MexAB system. The obtained analysis of amino acid substitutions in TolC revealed that adaptation to the heterogenous efflux pump was conferred by substitutions of amino acyl residues located in the lower α-helical barrel in the periplasmic equatorial domain and the entrance coiled coils of TolC [47]. Protein sequence alignment of *E. coli* TolC versus that of *E. amylovora* TolC revealed that all the substituted amino acid residues reported by Bokma *et al*. [47] are conserved in both enterobacterial species (data not shown) thus by further substantiating a tight phylogenetic relatedness of AcrAB-TolC in *E. coli* and *E. amylovora*.

Interestingly, any complementation led to MIC wild type level of the species, into which the heterologous alleles were transferred. These results underscore the principle that antibiotics resistance is determined by both, efflux and outer membrane permeability. In this context, it is remarkable that permeability for many of the tested compounds differed between *E. amylovora* and *E. coli*.

### 2.4. In planta virulence assays

**Figure 1:**
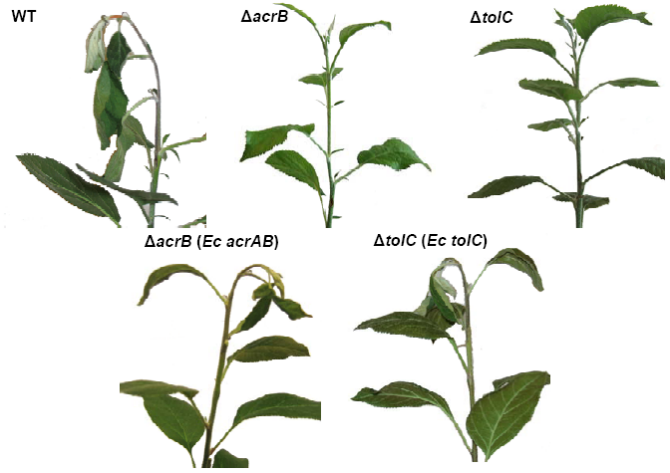
Pathogenicity assay on apple plants. Shoot tips were inoculated with 5 μL of 1 x 10^7^ CFU/mL bacterial suspensions. Disease symptoms such as the typical ‘shepherd’s crook’ formation and wilting developed on plants inoculated with *E. amylovora* wild type after one week of inoculation.

Previously it was demonstrated that *E. amylovora* mutants with defects in *acrB* and *tolC* were not causing fire blight symptoms on apple plants and showed significantly reduced *in planta* survival [33–34]. These mutants could be complemented by plasmid-borne homogenous alleles. Since heterogenous alleles of *acrB* and *tolC* led to wild type MIC values in the respective *E. amylovora* mutants *in vitro*, it was tested whether *acrB* and *tolC* of *E. coli* could restore virulence of *E. amylovora acrB* and *tolC* single mutants. Respective *E. amylovora* transformants were inoculated to apple plants using the mutants and the wild type as controls. Plant shoot tips were inoculated with defined numbers of bacterial cells by the so-called prick technique [48], which mimics the natural infection process. Typical fire blight symptoms in form of shepherd’s crook-like bending of the shoot tip after one week post inoculation as well as ooze formation and necrosis after three weeks post inoculation were induced by *E. amylovora* wild type and the *E. amylovora* mutants carrying *acrB* or *tolC* from *E. coli* in all 15 plants inoculated per strain without exceptions (Figure 1).

In contrast, the non-complemented mutants did not cause any disease symptoms thus confirming their previously reported *in planta* phenotypes [33–34]. All inoculated plants showed the same lack of symptom development. Interestingly, for the first time these results led to the conclusion that enterobacterial AcrAB-TolC hybrids consisting of mixed components from a plant pathogen and an intestinal species are sufficient to effectively combat plant defense reactions. These results furthermore substantiated our *in vitro* findings and showed that AcrB and TolC of *E. coli* and *E. amylovora* are apparently fully interchangeable. The herein obtained data are in line with recent findings of Krishnamoorthy *et al*. [49], who reported that the replacement of *E. coli* AcrB with its close homolog, MexB, from *Pseudomonas aeruginosa* formed a partially functional MDE system, AcrA-MexB-TolC, *in vitro* and that certain single amino acid substitutions in AcrA and MexB, respectively, were sufficient to render this MDE hybrid fully functional. The data obtained herein demonstrated that complementation of *acrAB* or *tolC* of a plant pathogen by respective alleles derived from *E. coli* can restore full virulence. In this respect, our results are in advancement towards those of Brown *et al*. [32], who had demonstrated that MDE components of the plant pathogen *R. solanacearum* were successfully complementing *E. coli* mutants *in vitro*.

## 3. Experimental

### 3.1. Bacterial strains, plasmids, and growth conditions

Bacterial strains and plasmids used in this study were listed in Tables 5 and 6. *E. coli* strains were routinely maintained at 37°C on Luria-Bertani (LB) medium, and *E. amylovora* strains were cultured at 28 °C on LB medium or asparagine minimal medium 2 (AMM2).

AMM2 has the following composition (per liter of deionized water): fructose, 10 g; l-asparagine, 4 g; Na_2_HPO_4_ × 7 H_2_O, 12.8 g; K_2_HPO_4_, 3 g; NaCl, 3 g; MgSO_4_ × 7 H_2_O, 0.2 g; nicotinic acid, 0.25 g; thiamine, 200 μg. *E. coli* DH5α was used as cloning host. Cultures were supplemented with 50 μg/mL ampicillin (Ap), 25 μg/mL chloramphenicol (Cm), 2 μg/mL gentamycin (Gm), and 25 μg/mL kanamycin (Km) when necessary. Bacterial growth was monitored using a spectrophotometer (OD at 600 nm).

### 3.2. Generation of tolC mutants in E. coli

*E. coli tolC*-deficient mutants TG1-1 and KAM3-1 were generated by marker exchange mutagenesis as follows. An 1851-bp fragment located in the *tolC* gene was PCR-amplified from *E. coli* TG1 using primer pair Ec-tolC-del-Fwd and Ec-tolC-del-Rev (Table 7). The PCR product was cloned into pGEM-T Easy (Promega, Mannheim, Germany) yielding plasmid pNK14. A 597-bp *Bss*HII fragment of pNK14 located in the *tolC* gene was replaced by a 1246-bp *Bss*HII fragment containing the gentamycin resistance cassette (Gm^r^) derived from pBBR1MCS-5 [52] yielding the 5515-bp plasmid pNK15. A 2500-bp *Eco*RI fragment cut from pNK15 was ligated into *Eco*RI-digested pCAM-MCS [33] yielding the final *tolC* replacement plasmid pNK16 (6207 bp). *E. coli* S17-1 *λ-pir* was used as host for the suicide plasmid. Plasmid pNK16 was transferred via electroporation into electro-competent cells of *E. coli* TG1 and its *acrB* mutant KAM3, which subsequently were grown at 37°C for 1 h in SOC broth and plated on LB containing gentamycin. Putative mutants were screened for homologous recombination events by checking their antibiotics resistance. To confirm the altered genotypes, PCR amplification of *tolC* using primers Ec-tolC-del-Fwd and Ec-tolC-del-Rev (Table 7) was carried out yielding a 2500-bp fragment for the *tolC* mutants as opposed to the presence of an 1851-bp fragment for the wild type.

### 3.3. Complementation of mutants

The plasmid for complementation of the *tolC*-deficient *E. coli* was generated as follows: a 2012-bp fragment containing the *tolC* region was PCR amplified from *E. coli* TG1 using primer pair Ec-tolC-com-Fwd and Ec-tolC-com-Rev (Table 7). The PCR fragment was cloned into *Spe*I-*Sac*I-digested pBBR1MCS [52] yielding the *E. coli tolC* complementation plasmid pNK17 (6719 bp). Plasmid pNK7 [34] was used to complement the *tolC* mutant of *E. amylovora*.

The plasmids for complementation of the *acrAB*- and *acrABR*-deficient *E. coli* mutants were generated as follows: 4701-bp and 5596-bp fragments containing the *acrAB* and *acrAB-R* regions, respectively, were PCR amplified from *E. coli* TG1 using the primer pairs Ec-acr-com-Fwd/Ec-acr-com-Rev- and Ec-acr-com-Fwd/Ec-acr-com-Rev+ (Table 7). The PCR fragments were cloned into *Hind*III-*Sac*I-digested pBBR1MCS [52] yielding the *E. coli acrAB* complementation plasmid pNK18 (9408 bp) and the *E.coli acrAB-R* complementation plasmid pNK19 (10303 bp).

The plasmid for complementation of the *acrABR*-deficient *E. amylovora* mutant was generated as follows: a 5746-bp fragment containing the *acrAB-R* region was PCR amplified from *E. amylovora* 1189 using the primer pair Ea-acr-Com-Fwd+ and Ea-acr-Com-Rev (Table 7). The PCR fragment was cloned into *Spe*I-*Sac*I digested pBBR1MCS [52] yielding the *E. amylovora acrAB-R* complementation plasmid pNK20 (10453 bp). Plasmid pNK8 [34] was used to complement the *acrB* mutant of *E. amylovora*.

### 3.4. Drug susceptibility tests

The minimal inhibitory concentrations (MIC) were determined by a twofold dilution assay in Mueller-Hinton broth (MHB) (Becton Dickinson, Heidelberg, Germany) and AMM2, respectively. All tests were done in triplicate following the National Center for Clinical Laboratory Standards recommendations [53]. Chemicals were obtained from Sigma-Aldrich (Taufkirchen, Germany). *E. amylovora* strains were incubated at 28 °C, and *E. coli* strains were incubated at 37 °C. The MIC was defined as the lowest concentration of an antibiotic that completely stopped visible cell growth. Bacterial growth was examined by visual and photometric inspection after 24 h of incubation.

### 3.5. Plant materials and pathogenicity assay on apple

Apple plants (rootstock Malus MM106) were grown in a light chamber at 20 to 25 °C, 60% humidity, with a 12-h photoperiod (15,000 lux). *E. amylovora* strains were grown on LB agar plates for 48 h, re-suspended in sterile 0.9% NaCl solution, and diluted to a cell density of 1 x 10^7^ CFU/mL for inoculation. Apple plants were inoculated by the prick technique as described previously [48]. Each bacterial strain was inoculated into shoots of five individual plants by placing 5 μL of bacterial suspensions onto each wound on the shoot tip. The experiment was repeated three-fold. Plants were monitored for symptom development daily.

## 4. Conclusions

The AcrAB-TolC system is widely distributed among Gram-negative bacteria and represents a bacterial defense system not only against antibiotics, but also against numerous structurally unrelated molecules present in different ecological niches, such as bile salt in the intestinal tract and phytoalexins in plants. Herein it was demonstrated that components of the MDE system, AcrAB-TolC, of two different enterobacterial species are interchangeable despite of the significantly diverse ecological niches, in which both species live. On one hand, this might reflect the broad flexibility of this important bacterial antibiotics resistance determinant. On the other hand, these results suggested that habitat-borne divergence of antibiotics exposure did not lead to significant structural modifications of this MDE system during evolution. For the first time, it was demonstrated that respective *E. coli* genes can restore full virulence to *E. amylovora* mutants defective in the genes encoding for AcrAB-TolC.

## Figures and Tables

**Table 1:** Susceptibility of *Escherichia coli* strains to different compounds in complex medium MHB.

Compounds	MIC*a* (μg/mL)							
	TG1	Δ*acrB*	Δ*tolC*	Δ*acrB*	Δ*acrB*	Δ*acrB*	Δ*tolC*	Δ*tolC*
				Δ*tolC*	(*Ea acrAB*)	(*Ec acrAB*)	(*Ea tolC*)	(*Ec tolC*)
Phloretin	>1000	250	125	125	>1000	>1000	>1000	>1000
Naringenin	>1000	125	125	125	>1000	>1000	>1000	>1000
(+)-Catechin	>1000	250	250	250	>1000	>1000	>1000	>1000
Quercetin	>1000	125	62.5	62.5	>1000	>1000	>1000	>1000
Berberine	>1000	62.5	62.5	62.5	>1000	>1000	>1000	>1000
Bile salt	>1000	15.6	15.6	15.6	>1000	>1000	>1000	>1000

Acriflavine	15.6	1.56	1.56	1.56	31.2	31.2	15.6	15.6
Novobiocin	1000	50	50	25	>1000	>1000	1000	1000
Ampicillin	31.2	6.25	6.25	6.25	31.2	31.2	31.2	31.2
Cefoperazone	12.5	0.75	0.75	0.75	6.25	6.25	6.25	12.5
Mitomycin	50	2.5	1.25	1.25	50	100	50	50
Tetracycline	3.12	0.15	0.075	0.075	3.12	3.12	3.12	3.12
Nalidixic acid	10	1	1	1	10	10	5	10
Norfloxacin	0.62	0.062	0.062	0.062	0.62	0.62	0.62	1.25
Ciprofloxacin	0.62	0.062	0.062	0.062	0.62	0.62	0.62	0.62
Amikacin	1.25	1.25	0.15	0.15	1.25	1.25	2.5	1.25
Tobramycin	2.5	1.25	0.15	0.075	2.5	5	5	5
Erythromycin	12.5	3.12	0.62	0.15	12.5	25	25	12.5
Rifampin	12.5	1.56	0.31	0.075	25	25	12.5	25
Jasmone	500	125	31.2	7.5	500	1000	1000	250
Clotrimazole	62.5	6.25	1.56	0.31	62.5	31.2	31.2	62.5

SDS	1000	100	100	100	1000	1000	1000	1000
Ethidium bromide	250	15.6	15.6	15.6	250	500	125	250
Crystal violet	12.5	2.5	2.5	2.5	12.5	25	12.5	12.5

^a^ MIC determination by the dilution assay was repeated at least three times in each case thereby confirming consistencies of MIC values.

**Table 2:** Susceptibility of *Erwinia amylovora* strains to different compounds in complex medium MHB.

Compounds	MIC*a*(μg/mL)							

	1189	Δ*acrB*	Δ*tolC*	Δ*acrB*	Δ*acrB*	Δ*acrB*	Δ*tolC*	Δ*tolC*
				Δ*tolC*	(*Ea acrAB*)	(*Ec acrAB*)	(*Ea tolC*)	(*Ec tolC*)
Phloretin	1000	125	125	125	>1000	>1000	>1000	>1000
Naringenin	1000	62.5	125	125	1000	1000	1000	1000
(+)-Catechin	>1000	125	250	250	>1000	>1000	>1000	>1000
Quercetin	1000	62.5	62.5	125	>1000	>1000	1000	1000
Berberine	1000	31.2	31.2	31.2	1000	1000	1000	1000
Bile salt	>1000	125	125	125	>1000	>1000	>1000	>1000

Acriflavine	15.6	1.56	3.12	3.12	31.2	31.2	31.2	31.2
Novobiocin	62.5	1.56	1.56	1.56	62.5	31.2	62.5	62.5
Ampicillin	62.5	6.25	12.5	6.25	62.5	62.5	62.5	31.2
Cefoperazone	12.5	3.12	3.12	3.12	12.5	25	12.5	12.5
Mitomycin	6.25	0.31	0.62	0.62	12.5	25	12.5	12.5
Tetracycline	6.25	0.62	0.62	0.62	12.5	6.25	6.25	12.5
Nalidixic acid	1.25	0.12	0.12	0.12	1.25	0.62	1.25	1.25
Norfloxacin	0.62	0.031	0.062	0.062	0.62	0.62	0.62	0.62
Ciprofloxacin	0.62	0.062	0.062	0.062	0.62	0.62	0.62	0.62
Amikacin	2.5	2.5	0.31	0.31	1.25	1.25	2.5	1.25
Tobramycin	2.5	1.25	0.075	0.15	2.5	2.5	2.5	5
Erythromycin	12.5	3.12	0.62	0.15	12.5	25	25	12.5
Rifampin	12.5	3.12	0.31	0.075	25	12.5	12.5	25
Jasmone	250	62.5	15.6	3.12	250	250	500	500
Clotrimazole	15.6	3.12	0.15	0.075	62.5	31.2	62.5	31.2

SDS	>1000	100	100	100	>1000	>1000	1000	1000
Ethidium bromide	31.2	3.12	3.12	3.12	62.5	62.5	62.5	62.5
Crystal violet	3.12	0.62	0.62	0.62	3.12	3.12	3.12	6.25

^a^ MIC determination by the dilution assay was repeated at least three times in each case thereby confirming consistencies of MIC values.

**Table 3:** Susceptibility of *Escherichia coli* strains to different compounds in minimal medium AMM2.

Compounds	MIC *a* (μg/mL)							
	TG1	Δ*acrB*	Δ*tolC*	Δ*acrB*	Δ*acrB*	Δ*acrB*	Δ*tolC*	Δ*tolC*
				Δ*tolC*	(*Ea acrAB*)	(*Ec acrAB*)	(*Ea tolC*)	(*Ec tolC*)
Phloretin	>1000	250	125	250	>1000	>1000	>1000	>1000
Naringenin	>1000	125	250	125	>1000	>1000	>1000	>1000
(+)-Catechin	>1000	125	125	62.5	>1000	>1000	>1000	>1000
Quercetin	>1000	125	125	62.5	>1000	>1000	>1000	>1000
Berberine	>1000	62.5	62.5	62.5	>1000	>1000	>1000	>1000
Bile salt	>1000	15.6	15.6	15.6	>1000	>1000	>1000	>1000

Acriflavine	7.5	1.56	1.56	1.56	7.5	15.6	7.5	15.6
Novobiocin	1000	100	100	50	>1000	>1000	>1000	1000
Ampicillin	15.6	3.12	6.25	3.12	31.2	31.2	31.2	31.2
Cefoperazone	6.25	0.62	0.62	0.62	6.25	6.25	6.25	12.5
Mitomycin	12.5	1.5	1.5	1.5	12.5	12.5	12.5	12.5
Tetracycline	1.56	0.15	0.15	0.075	1.56	1.56	3.12	3.12
Nalidixic acid	6.25	1.25	0.62	0.62	12.5	6.25	12.5	12.5
Norfloxacin	0.62	0.062	0.062	0.062	0.62	0.62	0.62	1.25
Ciprofloxacin	0.62	0.015	0.062	0.062	0.62	0.62	0.62	0.62
Amikacin	1.25	1.25	0.15	0.075	1.25	1.25	2.5	1.25
Tobramycin	1.25	1.25	0.075	0.075	2.5	5	5	5
Erythromycin	25	3.12	0.62	0.15	12.5	25	12.5	12.5
Rifampin	12.5	1.56	0.31	0.075	25	25	12.5	25
Jasmone	500	125	31.2	7.5	1000	500	1000	500
Clotrimazole	62.5	6.25	1.56	0.31	125	62.5	125	62.5

SDS	500	100	100	100	500	500	500	1000
Ethidium bromide	125	1.56	1.56	1.56	250	250	125	250
Crystal violet	6.25	1.25	0.62	0.62	6.25	6.25	6.25	12.5

^a^ MIC determination by the dilution assay was repeated at least three times in each case thereby confirming consistencies of MIC values.

**Table 4:** Susceptibility of *Erwinia amylovora* strains to different compounds in minimal medium AMM2.

Compounds	MIC*a*(μg/mL)							
	1189	Δ*acrB*	Δ*tolC*	Δ*acrB*	Δ*acrB*	Δ*acrB*	Δ*tolC*	Δ*tolC*
				Δ*tolC*	(*Ea acrAB*)	(*Ec acrAB*)	(*Ea tolC*)	(*Ec tolC*)
Phloretin	1000	125	125	125	>1000	>1000	>1000	>1000
Naringenin	1000	62.5	125	125	1000	1000	1000	1000
(+)-Catechin	>1000	125	250	250	>1000	>1000	>1000	>1000
Quercetin	1000	62.5	62.5	125	>1000	>1000	1000	1000
Berberine	1000	31.2	31.2	31.2	1000	1000	1000	1000
Bile salt	>1000	125	125	125	>1000	>1000	>1000	>1000

Acriflavine	15.6	1.56	3.12	3.12	31.2	31.2	31.2	31.2
Novobiocin	62.5	1.56	1.56	1.56	62.5	31.2	62.5	62.5
Ampicillin	62.5	6.25	12.5	6.25	62.5	62.5	62.5	31.2
Cefoperazone	12.5	3.12	3.12	3.12	12.5	25	12.5	12.5
Mitomycin	6.25	0.31	0.62	0.62	12.5	25	12.5	12.5
Tetracycline	6.25	0.62	0.62	0.62	12.5	6.25	6.25	12.5
Nalidixic acid	1.25	0.12	0.12	0.12	1.25	0.62	1.25	1.25
Norfloxacin	0.62	0.031	0.062	0.062	0.62	0.62	0.62	0.62
Ciprofloxacin	0.62	0.062	0.062	0.062	0.62	0.62	0.62	0.62
Amikacin	1.25	1.25	0.075	0.075	1.25	2.5	1.25	1.25
Tobramycin	2.5	2.5	0.15	0.15	2.5	2.5	2.5	2.5
Erythromycin	25	6.25	1.25	0.31	12.5	25	12.5	12.5
Rifampin	6.25	1.56	0.15	0.075	25	25	12.5	25
Jasmone	125	31.2	7.5	1.56	250	250	250	250
Clotrimazole	31.2	6.25	3.12	0.31	62.5	31.2	31.2	62.5

SDS	>1000	100	100	100	>1000	>1000	1000	1000
Ethidium bromide	31.2	3.12	3.12	3.12	62.5	62.5	62.5	62.5
Crystal violet	3.12	0.62	0.62	0.62	3.12	3.12	3.12	6.25

^a^ MIC determination by the dilution assay was repeated at least three times in each case thereby confirming consistencies of MIC values.

**Table 5:** *E. coli* and *E. amylovora* strains used in this study.

Strain	Relevant characteristics	Reverence or source
*E. coli*		[42]
DH5α	*supE*44 Δ*lac*U169 (φ80 *lacZ* ΔM15) *hsdR*17 *recA*1 *endA*1 *gyrA*96 *thi*-1	
S17-1 *λ-pir*	*λ-pir* lysogen of S17-1	[50]
TG1	*subE hsd*Δ5 *thi* Δ(*lac-proAB*) F`[*traD*36 *proAB*^+^*lacI^q^lacZ*ΔM15]	[42]
KAM3	*acrB* mutant of TG1	[51]
TG1-1	Gm^r^, *tolC* mutant of TG1	This study
KAM3-1	Gm^r^, *tolC* mutant of KAM3	This study
*E. amylovora*		
1189	wild type	GSPB^a^
1189-3	Km^r^, *acrB* mutant of 1189	[33]
1189-25	Gm^r^, *tolC* mutant of 1189	[34]
1189-3-3	Km^r^, Gm^r^, *acrB tolC* mutant of 1189	[34]

^a^ GSPB, Göttinger Sammlung phytopathogener Bakterien, Göttingen, Germany.

**Table 6:** Plasmids used in this study.

Plasmid	Relevant characteristics	Reference or source
pGEM-T Easy	Ap^r^, ColE1 origin	Promega
pNK14	Ap^r^, 1851-bp PCR fragment containing *E. coli tolC* in pGEM-T Easy	This study
pBBR1MCS-5	Gm^r^, Source of the Gm^r^ cassette	[52]
pNK15	Ap^r^, replacement of a 597-bp BssHII fragment from the *tolC* gene of pNK14 with a 1246-bp fragment containing the Gm^r^ cassette from pBBR1MCS-5	This study
pCAM-MCS	Ap^r^, pCAM140-derivative without mini-Tn5, contains the MCS of pBluescript II SK (+)	[33]
pNK16	Ap^r^, Gm^r^, 2500-bp EcoRI fragment from pNK15 ligated into EcoRI-linearized pCAM-MCS	This study
pBBR1MCS	Cm^r^, ColE1 origin	[52]
pNK17	Cm^r^, 2012-bp SpeI-SacI fragment carrying *tolC* of *E. coli* cloned in pBBR1MCS in opposite direction of *lacZ*	This study
pNK18	Cm^r^, 4701-bp HindIII-SacI fragment carrying *acrAB* of *E. coli* cloned in pBBR1MCS in opposite direction of *lacZ*	This study
pNK19	Cm^r^, 5596-bp HindIII-SacI fragment carrying *acrAB-R* of *E. coli* cloned in pBBR1MCS in opposite direction of *lacZ*	This study
pNK7	Cm^r^, 2630 bp SpeI-SacII fragment carrying *tolC* of *E. amylovora* cloned in pBBR1MCS in opposite direction of *lacZ*	[34]
pNK8	Cm^r^, 4917 bp SpeI-SacI fragment carrying *acrAB* of *E. amylovora* cloned in pBBR1MCS in opposite direction of *lacZ*	[34]
pNK20	Cm^r^, 5746 bp SpeI-SacI fragment carrying *acrAB-R* of *E. amylovora* cloned in pBBR1MCS in opposite direction of *lacZ*	This study

**Table 7:** Oligonucleotide primers used in this study.

Primer	Nucleotide sequence (5'–3')*
Ec-tolC-del-Fwd	AACTTCATCACGCACTGG
Ec-tolC-del-Rev	TTGCTGAACGACTGGTGC
Ec-tolC-com-Fwd	GATGAGCTCAACTTCATCACGCACTGG
Ec-tolC-com-Rev	CGAACTAGTATAGAGGATGGCTGGTCG
Ec-acr-com-Fwd	CGATAAGCTTGAGATCCTGAGTTGGTGG
Ec-acr-com-Rev-	GCTAGAGCTCAGCGAGGTGGATGATACC
Ec-acr-com-Rev+	GCTAGAGCTCGTATCTGTCAGATCCTGC
Ea-acr-com-Fwd+	CGAGAGCTCGAATACGGTTCTCTGAGC
Ea-acr-com-Rev	GATACTAGTCGGTATAGTAAACGTGCG

* Restriction sites incorporated into primers are underlined: AAGCTT, *Hind*III; GAGCTC, *Sac*I; and ACTAGT, *Spe*I.
